# Co-crystallization of an organic solid and a tetraaryladamantane at room temperature

**DOI:** 10.3762/bjoc.17.103

**Published:** 2021-06-21

**Authors:** Fabian Rami, Jan Nowak, Felix Krupp, Wolfgang Frey, Clemens Richert

**Affiliations:** 1 Institute of Organic Chemistry, University of Stuttgart, Pfaffenwaldring 55, 70569 Stuttgart, Germany

**Keywords:** adamantanes, crystallization, organic solids, structure elucidation, X-ray crystallography

## Abstract

Tetraaryladamantanes have proven useful as chaperones for the co-crystallization of small molecules that do not readily crystallize by themselves. The co-crystals are often useful for structure elucidation. Usually, the small molecules are encapsulated in the crystal lattice of the aryladamantane that forms during rapid thermal crystallization. Thus far, co-crystallization has been limited to liquids as guest molecules. Here we report the co-crystal structures of phenol, which is solid at room temperature, with both 1,3,5,7-tetrakis(2,4-dimethoxyphenyl)adamantane (TDA) and 1,3,5,7-tetrakis(2,4-diethoxyphenyl)adamantane (TEO). The co-crystals were obtained from solutions in dichloromethane by slow evaporation or diffusion. The implications for generating other co-crystals of two solids are briefly discussed.

## Introduction

Obtaining a crystal suitable for X-ray crystallography can be a challenge for organic compounds, and crystallization continues to be as much of an art as a science. Crystal engineering is a burgeoning field [1], but it continues to be challenging to predict the crystal system in which a compound will crystallize [2], the rate at which crystallization will occur, or the likelihood that crystallization will produce a solvate or pure crystals of the compound of interest alone [3]. Furthermore, many organic compounds continue to resist attempts to crystallize them [4], either because they remain liquid at the given temperature, or because they form glassy or amorphous solids instead. It is often unclear whether crystallization is too slow or too unfavorable to occur.

There are several approaches to overcome the reluctance of some organic compounds to crystallize. Among them are the crystallization of small molecules at very low temperatures [5–7], and the diffusion into a crystal lattice set up by a larger compound or crystalline complex [8–9]. For some medium-size molecules, supercritical fluids have been used to obtain co-crystals [10]. Other co-crystals were obtained by encapsulating guest molecules in pre-planned frameworks, set up with organic salts [11]. At this point in time, there is no method that can be considered universal, and there is room for new approaches.

We have recently described encapsulating organic crystals (EnOCs) as a class of organic crystals that are formed with a broad range of small molecules as guests and tetraaryladamantanes as hosts. The encapsulation occurs even though the host compound assembling into the crystal lattice is able to also crystallize in solvate-free form [12]. Three tetraaryladamantanes (TAAs) were found to show this behavior as hosts, namely 1,3,5,7-tetrakis(2,4-dimethoxyphenyl)adamantane (TDA), 1,3,5,7-tetrakis(2,4-diethoxyphenyl)adamantane (TEO), and 1,3,5,7-tetrakis(2-bromo-4-methoxyphenyl)adamantane (TBro). The X-ray crystal structures of over 100 EnOCs have been reported thus far [13–16], including structures with chiral guests that allow for the determination of the absolute [17] or relative configuration [18]. One limitation of the EnOC method was that the guest compound to be encapsulated had to be a liquid, so that it could act as solvent for the TAA, which would rapidly crystallize upon cooling of a hot, saturated solution. Here we report two co-crystal structures of TAAs with phenol, obtained by crystallization at room temperature, using dichloromethane as solvent.

## Results

**Figure 1:**
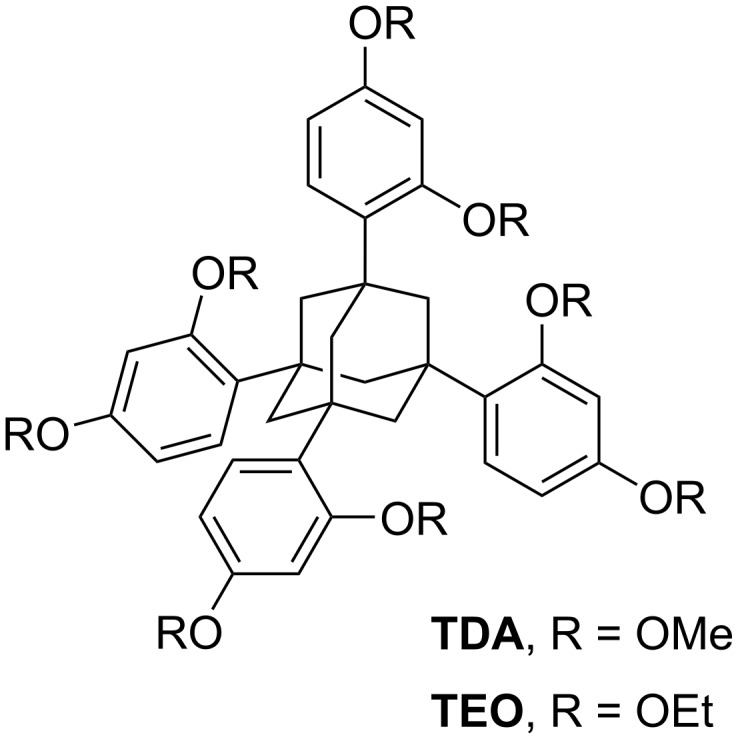
Structure of TDA and TEO, the crystallization hosts used for co-crystallization.

We opted for a benzene derivative for our first foray into organic solids to be encapsulated in TAA crystals, because a number of benzene derivatives have been found in EnOCs in the past [13–15]. Phenol was considered an interesting case, as the molecule crystallizes easily; setting up what may be considered a molecular competition between two readily crystallizing compounds (TAAs usually crystallize within minutes upon cooling of a saturated solution). Phenol is also inexpensive and the parent compound of a wide range of chemically interesting molecular entities, further favoring it as test substance. Two molecular hosts were to be employed, namely TDA and TEO (Figure 1), both of which are readily synthesized in high yield [14–15].

Exploratory experiments were performed aiming at crystallizing at elevated temperature using the thermal crystallization approach [17]. However, achieving a high end temperature of the temperature gradient met with technical difficulties and the approach was further complicated by the high solubility of the TAAs tested in the phenol melts. This prompted us to test a more conventional approach, using dichloromethane as solvent and slow evaporation or diffusion of a poor solvent into the dichloromethane solution to obtain co-crystals.

**Figure 2:**
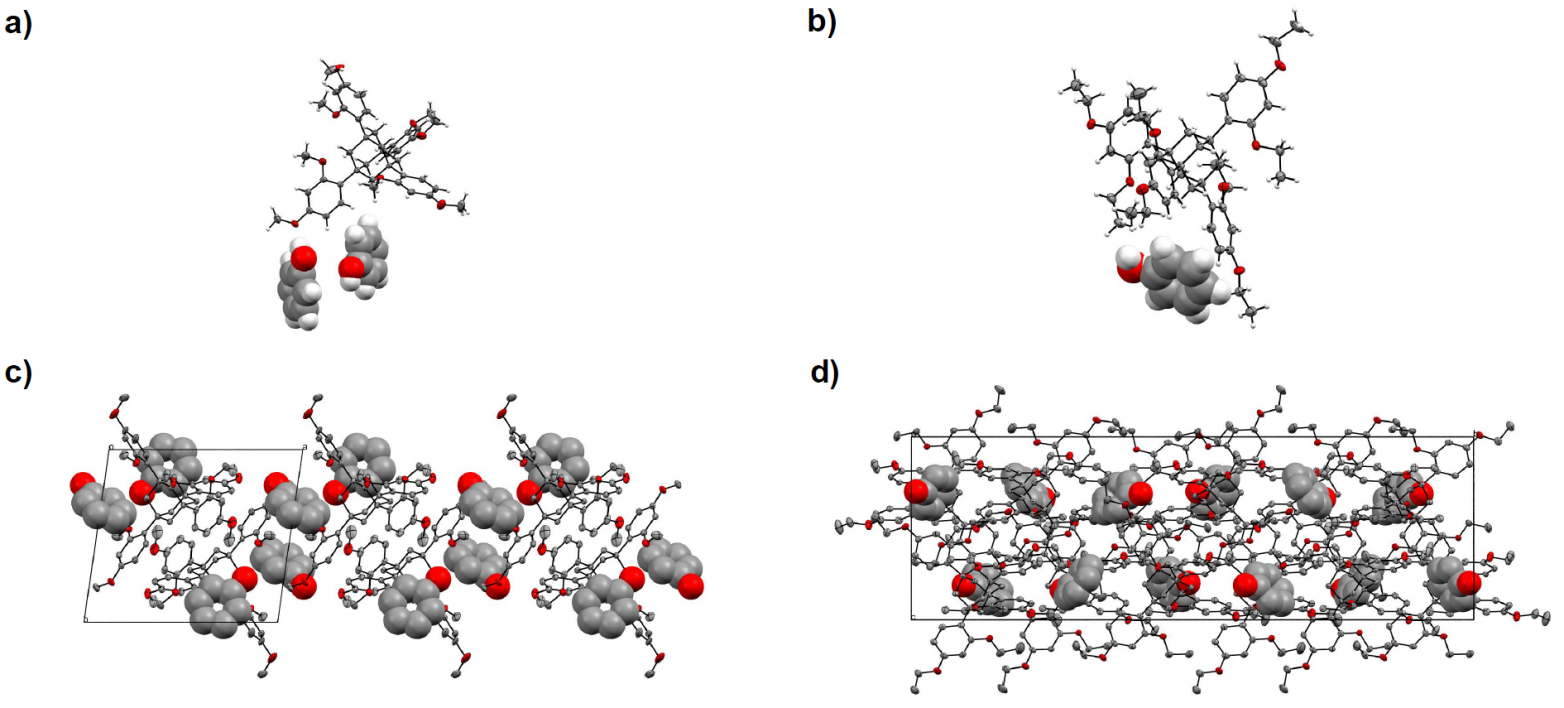
Details of the X-ray crystal structures obtained, shown as ORTEP plots at 50% probability, with van der Waals radii of the phenol molecules set to 60%. a) Asymmetric unit of the TDA/phenol co-crystal (space group *P*-1). b) Portion of the asymmetric unit of the TEO/phenol co-crystal (space group *Cc*). c) Crystal packing in the TDA/phenol system, and d) crystal packing in the TEO/phenol system.

Two such solvent-based crystallization runs produced the co-crystal structures shown in Figure 2. For TDA, 5 mg of the chaperone and 1 mg of phenol were dissolved in dichloromethane, followed by placing a layer of *n*-decane on top. For TEO, 5 mg of the TAA and 50 mg of phenol were dissolved in CH_2_Cl_2_, and the solvent was allowed to slowly evaporate through a small-gauge needle. In either case, crystal growth was slow, and crystals suitable for X-ray crystallography were collected after more than one week.

Table 1 lists the details of the structures observed. In case of the TDA/phenol co-crystals (Figure 2a/c), the asymmetric unit is made up of one host and two guest molecules, so the overall stoichiometric ratio is 1:2 (TDA/phenol). One methyl substituent of the chaperone was found to be disordered, but otherwise well resolved structures were obtained, with hydrogen bonds between phenol hydroxy groups and ether groups of the chaperone (see Table S1 in Supporting Information File 1).

**Table 1:** Details of crystal structures solved.

entry	host	crystal system	space group	volume of u.c. [Å^3^]	*Z*^a^	density [Mg/m^3^]	molar ratio TAA:PhOH	*R1*^b^

1	TDA	triclinic	*P*-1	2310.6(3)	2	1.249	1:2	0.0554
2	TEO	monoclinic	*Cc*	14855.7(15)	12	1.190	1:1	0.0432

^a^Number of molecules in the unit cell, asymmetric unit; ^b^final *R* indices [*l* > 2 *σ*(*l*)]

For TEO, the crystal system of the co-crystals is monoclinic. The TEO/phenol asymmetric unit consists of three TEO and three phenol molecules, resulting in a molar ratio TEO/phenol of 1:1 (Figure 2b/d). In the crystals, the ethoxy group of one of the TAA molecules is disordered, and one phenol molecule exhibits a 120°-rotational disorder for its hydroxy group. But again, hydrogen bonds between phenol hydroxy groups and the alkoxy substituent of the crystallization chaperone can be resolved.

So, in both co-crystal lattices, hydrogen bonding stabilizes the packing arrangement. For TDA/phenol, the host builds the crystal lattice with TDA molecules in close proximity to each other and the phenol guest occupies cavities (Figure 2c). In comparison, possibly due to the steric demand of the ethoxy groups of TEO, an arrangement with alternating TEO and phenol molecules is found for the larger chaperone (Figure 2d).

## Discussion

Crystallization is one of the most important methods for purifying organic compounds in industry [19]. It usually produces solids made up of one compound only. It is common knowledge among synthetic chemists that mixtures are difficult to crystallize. In fact, crystallization to obtain single crystals that are suitable for X-ray crystallography is typically attempted only after some pre-purification has been performed. This highlights what challenges have to be met when trying to co-crystallize two solids.

The situation is different for solvents as the second component. Solvates are not uncommon [1]. However, obtaining a solvate is also different from inducing two solids to co-crystallize, as the solvent does not have the propensity to form its own crystal lattice at the chosen temperature.

Stepping back, one may ask what the difficulties are that usually preclude the formation of co-crystals. The difficulties probably have both kinetic and thermodynamic aspects. Kinetically, when crystallizing from a solvent, the solvent may be more likely to be encapsulated than the second solid-forming compound, as the solvent will be smaller and more abundant, making it the more likely molecule to be entrapped in cavities left by the scaffold-forming compound. The situation will be different, when there is shape complementarity between the two solids or when other specific interactions favor a co-crystal [20]. Thermodynamically, it is not unlikely that two separate sets of pure crystals, made up of only one organic compound each, represent the lower minimum in free energy, with both solvent and the competing organic molecule being eliminated during crystallization [20].

Thermal crystallization, induced by cooling a saturated solution of a TAA in the liquid guest molecule, is what has been used thus far by us to produce EnOCs. We suspected that the encapsulation of the guest molecules in the crystals occurs as a kinetic phenomenon [17]. The TAA finds a crystalline arrangement quickly, without full desolvation, and once the crystal lattice has formed, the guest molecule is unable to escape from it, blocking the path to solvent-free crystals that may be thermodynamically more favorable. As noted above, tightly packed solvent-free crystal systems exist for TAAs [12].

**Figure 3:**
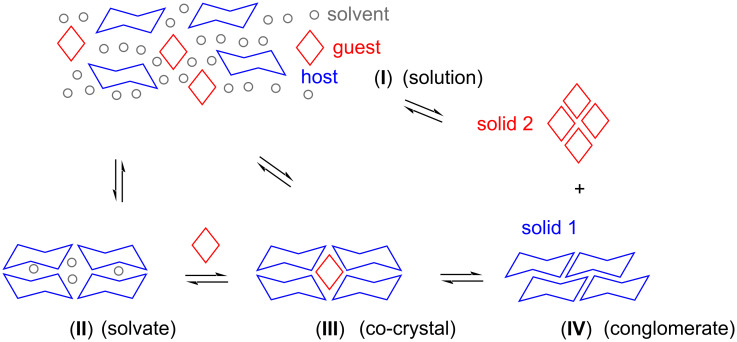
Possible states of two organic compounds capable of crystallizing at room temperature in a solvent. Colors used are blue for the chaperone, red for the smaller solid-forming organic compound, and gray for the solvent.

So, what is the most likely process underlying the co-crystal formation observed in our current study? Figure 3 shows possible molecular situations in cartoon format. We initially suspected that a slow crystallization, induced by evaporation or diffusion, would make it unlikely to obtain co-crystals, as the long time intervals involved should favor a thermodynamic, rather than a kinetic product. In other words, starting from a solution (**I**), we expected to find a conglomerate (**IV**), not co-crystals (**III**). This was not what was found experimentally.

The two X-ray crystal structures (Figure 2, Table 1) show that slow crystallization does not preclude the formation of co-crystals. One reason for this may be that TAA solvates encapsulating dichloromethane can be metastable [13]. For example, leaving them exposed to air leads to loss of the solvent and a brittle material that slowly loses its crystalline order. Solvates (**II**) may thus have played a role as intermediates. They may have formed while the dichloromethane content was still high, but may have given way to co-crystals as more and more of the DCM evaporated. Changes in the content of guest molecules without loss of macroscopically visible crystallinity have been observed before [13].

Independent of what the pathway to co-crystallization was in the present case, the slow crystallization with solvent, as compared to the rapid thermal crystallization with a liquid guest alone, is not in conflict with the notion that co-crystallization suffers from processes that inhibit nucleation or crystal growth. Co-crystals are still expected to form slowly because of the low probability of molecules arriving at the growing crystallites in the 'correct order of events'.

We note that our approach is focused on obtaining single crystals suitable for X-ray crystallographic analysis. We study analytes much smaller than the chaperone. Elegant works by Aakeröy, MacGillivray and others have shown how designed co-crystals may be obtained from organic molecules chosen for their shape complementarity and ability to engage in specific molecular interactions [21–22]. Such co-crystals are a fascinating class of materials with applications very different from mere structure elucidation.

## Conclusion

Taken together, the structures presented here suggest that even for readily crystallizing organic compounds co-crystals can be the favored outcome of crystallization from a solution in a good, but volatile solvent. This makes it interesting to pursue co-crystallization as a means to obtain crystals of other organic solids that have thus far evaded crystallization by themselves.

## Supporting Information

File 1 Materials and methods, protocol for the synthesis of TEO, crystallization protocols, and additional data for crystal structures.
