# Supplementary material for: Co-crystallization of an organic solid and a tetraaryladamantane at room temperature
Source: Beilstein J Org Chem. 2021 Jun 21;17:1476–80. doi: 10.3762/bjoc.17.103 (PMC8239258; doi:10.3762/bjoc.17.103)
Supplement: File 1 — Materials and methods, protocol for the synthesis of TEO, crystallization protocols, and additional data for crystal structures. [file Beilstein_J_Org_Chem-17-1476-s001.pdf]

BEILSTEIN JOURNAL OF ORGANIC CHEMISTRY

Supporting Information

for

Co-crystallization of an organic solid and a tetraaryladamantane at room temperature

Fabian Rami, Jan Nowak, Felix Krupp, Wolfgang Frey and Clemens Richert

Beilstein J. Org. Chem. **2021,** *17,* 1476–1480. doi:10.3762/bjoc.17.103

Materials and methods, protocol for the synthesis of TEO, crystallization protocols, and additional data for crystal structures

License and Terms: This is a supporting information file under the terms of the Creative Commons Attribution License (https://creativecommons.org/licenses/by/4.0). Please note that the reuse, redistribution and reproduction in particular requires that the author(s) and source are credited and that individual graphics may be subject to special legal provisions.
The license is subject to the *Beilstein Journal of Organic Chemistry* terms and conditions: (https://www.beilstein-journals.org/bjoc/terms)

Table of contents

1. Materials and methods S1

2. X-ray crystal structures.. S5

3. References S7

1. Materials and methods

Chemicals. All chemicals were obtained from commercial suppliers and were used without further purification. 1,3,5,7-Tetrakis(2,4-dimethoxyphenyl)adamantane (TDA) as crystallization chaperone was synthesized according to a literature-known method[S1] that proceeds via 1,3,5,7-tetrabromoadamantane and 1,3,5,7-tetrahydroxyadamantane as intermediates.[S2] 1,3,5,7-Tetrakis(2,4-diethoxyphenyl)adamantane (TEO) was obtained using a slight modification of a known procedure, as detailed below. Tetraaryladamantane chaperones are also available for sale by Bruker AXS GmbH (Karlsruhe, Germany).

For column chromatography, silica gel 60 (0.040–0.063 mm, 230–400 mesh) from Macherey-Nagel was used. Precoated ALUGRAM Xtra Sil G/UV254 (silica on aluminium) sheets from Macherey-Nagel were used for TLC. Eluent mixtures are given as volumetric ratios. NMR spectra were recorded on a Bruker Avance 300 spectrometer at 300 MHz (^{1}H NMR). Chemical shifts are reported in ppm and referenced to the residual solvent peak ($CDCl_3$: 7.26 ppm). Multiplet splitting J are reported in hertz (Hz) and signal shapes are abbreviated as follows: m (multiplet), s (singlet), t (triplet).

Preparation of 1,3,5,7-Tetrakis(2,4-diethoxyphenyl)adamantane (TEO). A suspension of 1,3,5,7-adamantanetetraol (50.0 mg, 0.25 mmol, 1.0 equiv) in 1,3-diethoxybenzene (3.30 mL, 20.00 mmol, 80.0 equiv) in a 10 mL round-bottomed flask with an NS14.5 ground joint, where the neck was covered with aluminium foil, was heated to 60 °C. At this temperature, *p*-toluenesulfonic acid monohydrate (47.5 mg, 0.25 mmol, 1.0 equiv) was added. The reaction mixture was subsequently heated in the open flask to 160 °C in a fume hood to allow water to escape. After 66 h, the reaction mixture was cooled to room temperature, diluted with dichloromethane (20 mL), and washed with a saturated aqueous sodium bicarbonate solution (15 mL). The aqueous layer was extracted with dichloromethane (1 × 10 mL). The combined organic layers were washed with diluted hydrochloric acid (2 N,

15 mL) and saturated aqueous sodium chloride solution (15 mL). After drying over sodium sulfate, the solvent was removed in vacuo. Residual 1,3-diethoxybenzene was removed by vacuum distillation. The solid residue was subjected to silica gel column chromatography (eluent: petroleum ether/dichloromethane 1:1 to 0:100 *v*/*v*). After evaporation of the solvent, the crude product was dissolved in the minimal amount of dichloromethane and precipitated by the addition of methanol (approx. the ten-fold volume of dichloromethane used). The solvent was removed with a syringe, and the solid washed twice with methanol. After drying under vacuum, the pure TEO was obtained as a colorless solid in a yield of 77.1 mg (0.10 mmol, 39%). We note that instead of petroleum ether/dichloromethane mixtures, petroleum ether/ethyl acetate 10:1 (*v*/*v*) can be used as eluent with identical results to facilitate the purification.

The analytical data were in good agreement with the literature.[S3] **^{1}H NMR** (300 MHz, $CDCl_3$) δ = 7.24-7.22 (m, 4 H), 6.46-6.40 (m, 8 H), 4.05-3.98 (m, 16 H), 2.46 (s, 12 H), 1.40 (t, J = 7.0 Hz, 12 H), 1.33 (t, J = 6.9 Hz, 12 H) ppm.

Crystallization experiments

Co-crystallization of phenol and TEO. A 1 mL sample vial (Rotilabo glass vials, Carl Roth) was charged with TEO (5 mg, 6.3 µmol) and phenol (50 mg, 0.53 mmol). The solids were dissolved in dichloromethane (0.1 mL), and the sample vial was closed with a cap. The cap was subsequently punctured with a cannula (0.4 × 20 mm). The cannula was not removed to allow for evaporation of the dichloromethane. The vial was kept at room temperature (at approx. 16–23 °C). A slow formation of small crystals in the liquid mixture was observed. Crystal growth was monitored until X-ray quality crystals were picked and subjected to diffractometry, as stated in the crystallography section.

Co-crystallization of phenol and TDA. A 1 mL sample vial (Rotilabo glass vials, Carl Roth) was charged with TDA (5.0 mg, 7.3 µmol) and phenol (1.0 mg, 10 µmol). The solids were dissolved in dichloromethane (0.1 mL), and subsequently layered with *n*-decane at room temperature. After three days, crystal formation was first detected and allowed to proceed until crystals suitable for X-ray crystallography were picked and measured as stated in the crystallography section.

Crystallography. X-ray diffraction was performed on a Bruker Kappa APEXII Duo diffractometer at temperatures of either 135 K or 140 K using molybdenum Kα (λ = 0.71073 Å). Cell refinement and data reduction were performed with the program package

SAINT[S4] and absorption reduction was done with SADABS.[S4] X-ray structures were solved with SHELXL97[S5] software by direct methods. Isotropic refinement (least-squares) was also carried out with SHELXL-2014/7, followed by anisotropic refinements on F2 of all non-hydrogen atoms. The H-atom positions were calculated geometrically with riding models. All crystallographic data of the reported co-crystals have been deposited at the Cambridge Crystallographic Data Centre (CCDC). For their CCDC numbers, please see the respective entry in section 2. The programs Mercury 2020.3.0,[S5] SHELXTL-plus and XP (Sheldrick, 2001) were used to draw the X-ray structures shown.[S6]

Table S1: Hydrogen bonds for s2834lm1 [Å and °] (D: donor, H: hydrogen, A: acceptor).

**D-H--A**	***d* (D-H)**	***d* (H-A)**	***d* (D-A)**	**angle (DHA)**
O(1X)-H(1X)--O(6B)#1	0.84	1.98	2.811(3)	168
O(1Y)-H(1Y)--O(2A)#2	0.84	1.98	2.813(3)	172
O(1Z)-H(1Z)--O(6C)#3	0.84	1.98	2.804(3)	169
O(1W)-H(1W)--O(2B)#4	0.84	2.02	2.85(1)	175

Symmetry transformations used to generate equivalent atoms:
#1 x+1/2,y-1/2,z+1 #2 x,-y+1,z-1/2 #3 x,-y+1,z+1/2 #4 x,y,z

Table S2: Hydrogen bonds for s2779lm [A and deg.] (D: donor, H: hydrogen, A: acceptor).

**D-H--A**	***d* (D-H)**	***d* (H-A)**	***d* (D-A)**	**angle (DHA)**
O(1X)-H(1X)--O(2)#1	0.96(3)	1.87(3)	2.816(2)	169(2)
O(1Y)-H(1Y)--O(4)#2	0.85(3)	1.891(35)	2.725(3)	168(3)

Symmetry transformations used to generate equivalent atoms:

#1 x,y,z #2 x+1,y+1,z

Additional description of the structures.

The TEO/phenol host–guest system crystallized with three TEO and three phenol molecules in the asymmetric unit of the non-centrosymmetric space group *Cc*. Establishing an inversion matrix, the structure was refined as inversion twin. A *p*-ethoxy moiety of one TEO host is disordered and one phenol guest molecule shows a 120-degree rotational disorder of the OH function. The hydroxy group of the phenol molecules acts as acceptor for a couple of intermolecular linear hydrogen bonds to the oxygen of the *p*-ethoxy moieties of TEO as donor to stabilize the organisation of the packing (Table S1).

The TDA/phenol host–guest system crystallized with one TDA and two phenol molecules in the asymmetric unit of the centrosymmetric space group *P*-1. One methyl group of a *p*-methoxy moiety of the TDA host is disordered. The hydroxy groups of the phenol molecules form linear intermolecular hydrogen bonds to the oxygen atoms of the methoxy moieties of the TDA (Table S2).

2. X-ray crystal structures

TEO with encapsulated PhOH

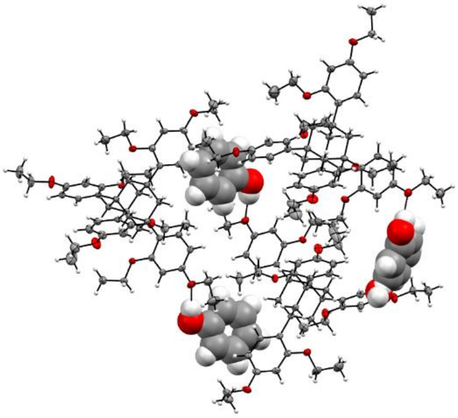

The X-ray structure is deposited at the Cambridge Crystallographic Data Centre (www.ccdc.cam.uk) under CCDC no. 2072012

Identification code	s2834lm1 (TEO/phenol 1:1)
Empirical formula	$C_{56}H_{70}O_9$
Formula weight	887.12
Temperature	140(2) K
Wavelength	0.71073 Å
Crystal system, space group	Monoclinic, Cc
Unit cell dimensions	a = 26.4235(13) Å, α = 90°
	b = 41.570(3) A, β = 133.603(2)°
	c = 18.6766(9) A, γ = 90°
Volume	14855.7(15) $Å^3$
Z, Calculated density	12, 1.190 Mg/m^3
Absorption coefficient	0.079 mm^{-1}
F(000)	5736
Crystal size	0.423 x 0.353 x 0.318 mm
Theta range for data collection	1.796 to 28.347°
Limiting indices	-35<=h<=35, -55<=k<=55, -24<=l<=24
Reflections collected / unique	183981 / 35092 [R(int) = 0.0348]
Completeness to theta = 25.242	100.0%
Absorption correction	Semi-empirical from equivalents
Max. and min. transmission	0.7457 and 0.7053
Refinement method	Full-matrix least-squares on F^2 (refined as inversion twin)
Data / restraints / parameters	35092 / 55 / 1822
Goodness-of-fit on F^2	1.036
Final R indices [I>2sigma(I)]	R1 = 0.0432, wR2 = 0.0882
R indices (all data)	R1 = 0.0746, wR2 = 0.0968
Absolute structure parameter	0.4(5) (inversion twin)
Extinction coefficient	n/a
Largest diff. peak and hole	0.366 and -0.235 $eÅ^{-3}$

TDA with encapsulated PhOH

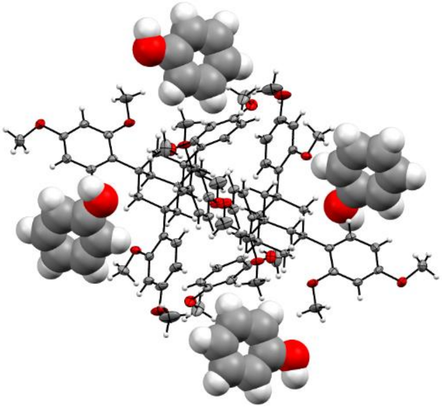

The X-ray structure is deposited at the Cambridge Crystallographic Data Centre (www.ccdc.cam.uk) under CCDC no. 2072014

Identification code	s2779lm (TDA/Phenol 1:2)
Empirical formula	$C_{54}H_{60}O_{10}$
Formula weight	869.02
Temperature	135(2) K
Wavelength	0.71073 Å
Crystal system, space group	Triclinic, P-1
Unit cell dimensions	a = 13.5810(12) Å, α = 118.207(2)°
	b = 13.7970(6) Å, β = 95.841(3)°
	c = 14.2159(6) Å, γ = 94.519(3)°
Volume	2310.6(3) $Å^3$
Z, Calculated density	2, 1.249 Mg/m^3
Absorption coefficient	0.085 mm^{-1}
F(000)	928
Crystal size	0.295 x 0.224 x 0.154 mm
Theta range for data collection	1.523 to 26.489°
Limiting indices	-16<=h<=17, -17<=k<=16, -17<=l<=17
Reflections collected / unique	36045 / 9528 [R(int) = 0.0473]
Completeness to theta = 25.242	99.9%
Absorption correction	Semi-empirical from equivalents
Max. and min. transmission	0.7454 and 0.7059
Refinement method	Full-matrix least-squares on F^2
Data / restraints / parameters	9528 / 8 / 604
Goodness-of-fit on F^2	1.035
Final R indices [I>2sigma(I)]	R1 = 0.0554, wR2 = 0.1132
R indices (all data)	R1 = 0.1124, wR2 = 0.1261
Extinction coefficient	n/a
Largest diff. peak and hole	0.420 and -0.387 e $Å^{-3}$

3. References

[S1] F. Krupp, W. Frey, C. Richert, *Angew. Chem. Int. Ed.* **2020**, *59*, 15875-15879, *Angew. Chem.* **2020**, *132*, 16009-16013.

[S2] A. Schwenger, W. Frey, C. Richert, *Chem. Eur. J.* **2015**, *21*, 8781-8789.

[S3] P.-E. Alexandre, A. Schwenger, W. Frey, C. Richert, *Chem. Eur. J.* **2017**, *23*, 9018-9021.

[S4] a) SAINT: Bruker **2012**, Bruker AXS Inc., Madison, Wisconsin, USA; b) SADABS: Bruker **2001**, Bruker AXS Inc., Madison, Wisconsin, USA.

[S5] a) C. F. Macrae, I. Sovago, S. J. Cottrell, P. T. A. Galek, P. McCabe, E. Pidcock, M. Platings, G. P. Shields, J. S. Stevens, M. Towler, P. A. Wood, *J. Appl. Cryst.* **2020**, **53**, 226-235; b) C. F. Macrae, I. J. Bruno, J. A. Chisholm, P. R. Edgington, P. McCabe, E. Pidcock, L. Rodriguez-Monge, R. Taylor, J. van de Streek and P. A. Wood, *J. Appl. Cryst.* **2008**, **41**, 466-470; c) C. F. Macrae, P. R. Edgington, P. McCabe, E. Pidcock, G. P. Shields, R. Taylor, M. Towler and J. van de Streek, *J. Appl. Cryst.* **2006**, **39**, 453-457; d) I. J. Bruno, J. C. Cole, P. R. Edgington, M. K. Kessler, C. F. Macrae, P. McCabe, J. Pearson and R. Taylor, *Acta Cryst.* **2002**, **B58**, 389-397; e) R. Taylor and C. F. Macrae, *Acta Cryst.* **2001**, **B57**, 815-827; f) G. M. Sheldrick, *Acta Cryst.* **2015**, **C71**, 3-8.

[S6] G. M. Sheldrick, *Acta Cryst.* **2008**, *A64*, 112-122.
